# Management of Uveitis-Related Choroidal Neovascularization: From the Pathogenesis to the Therapy

**DOI:** 10.1155/2014/450428

**Published:** 2014-04-27

**Authors:** Enzo D'Ambrosio, Paolo Tortorella, Ludovico Iannetti

**Affiliations:** Department of Ophthalmology, “Sapienza” University of Rome, Viale del Policlinico 155, 00161 Rome, Italy

## Abstract

Inflammatory choroidal neovascularization is a severe but uncommon complication of uveitis, more frequent in posterior uveitis such as punctate inner choroidopathy, multifocal choroiditis, serpiginous choroiditis, and Vogt-Koyanagi-Harada syndrome. Its pathogenesis is supposed to be similar to the wet age related macular degeneration: hypoxia, release of vascular endothelial growth factor, stromal cell derived factor 1-alpha, and other mediators seem to be involved in the uveitis-related choroidal neovascularization. A review on the factors implicated so far in the pathogenesis of inflammatory choroidal neovascularization was performed. Also we reported the success rate of single studies concerning the therapies of choroidal neovascularization secondary to uveitis during the last decade: photodynamic therapy, intravitreal bevacizumab, and intravitreal ranibizumab, besides steroidal and immunosuppressive therapy. Hereby a standardization of the therapeutic approach is proposed.

## 1. Introduction

Beside the well-known choroidal neovascularization (CNV) in the age related macular degeneration (ARMD) in myopic eyes or in angioid streaks, neovascular membranes can develop even as a complication of uveitis with an incidence of 2% [1], accounting for severe visual loss in patients with ocular infectious or noninfectious inflammatory diseases [2], also affecting young patients.

The prevalence of CNV secondary to uveitis varies among different entities, commonly occurring in presumed ocular histoplasmosis (POHS) (3.8%), toxoplasmosis, punctate inner choroidopathy (PIC) (17–40%), idiopathic multifocal choroiditis (MC) (33%), and serpiginous choroiditis (SC) [2]. Yet, CNV has been reported in up to 9% of patients with Vogt-Koyanagi-Harada disease (VKH) [1, 3].

## 2. Diagnosis

Inflammatory CNV can develop close to chorioretinal scar or choroidal granuloma and is classified topographically as foveal, juxtafoveal, or extrafoveal. The first is often early recognized by the patient himself complaining of metamorphopsia or central scotoma and can lead to the diagnosis of a subclinical posterior or intermediate uveitis. Otherwise, an extrafoveal membrane may be asymptomatic and can be found only at a follow-up examination or in case of posterior pole acute hemorrhage. As in ARMD, the microscopical features define type 1 or type 2 membrane, if it invades or not the subretinal space. In uveitis the classic membrane strongly shows the main type; it has a grayish appearance with an evidence of exudative or hemorrhagic foci surrounding the lesion. However, opthalmoscopically subretinal membrane could be missed because of very few levels of exudation; the only indirect sign could be a small intraretinal hemorrhagic lesion. Atrophic CNVs are yellow-white plaques. Often a bigger CNV can have a mixed pattern, bearing active foci in a globally fibrous plaque. A membrane can manifest only with macular edema or serous retinal detachment; however, macular edema and serous retinal detachment also can represent signs of inflammation found in course of intermediate/posterior uveitis, leading sometimes to a misdiagnosis or imprecise evaluation of the activity of the underlying disease.

In this case the role of the diagnostic imaging is crucial. Fluorescein angiography (FA) has been for long time the principal way to assess the presence and the activity of a CNV in uveitic patients, showing early hyperfluorescence in the choroidal phase and late leakage, associated sometimes with screen effect in presence of blood or pigment. Indocyanine green angiography (ICG) is useful for highlighting the feeder vessel or occult membranes; the new Heidelberg SLO video ICG enhances the diagnostic potentialities of this procedure. Nowadays a growing role is played by optical coherence tomography (OCT), a fast, noninvasive instrument able to assess the presence and the activity of the disease. Kotsolis et al. [4] showed that FA has a greater capability to detect the membrane features compared to OCT. But in the study of Kotsolis et al. [4] time domain OCT was used mostly; theoretically using the spectral domain OCT this discrepancy is unlikely to be observed, even though a definitive study still lacks.

## 3. Pathogenesis

Given the low incidence of inflammatory CNV and the difficulty in obtaining a reliable experimental model, most of our knowledge about this disease is mutated from the histopathological studies on ARMD-related CNV, supposing that similar clinical features correspond to common biological pathways.

In CNV a key role of vascular endothelial growth factor (VEGF) in the new blood vessels development has been widely demonstrated [5, 6].

VEGF is produced by endothelial cells, pericytes, Müller cells, Ganglion cells, photoreceptors, and RPE cells that can produce the growth factor in a polarized way towards Brüch's membrane and choriocapillaris [7, 8]. The major signal to the production of these cytokines seems to be the hypoxia via the activation of hypoxia induced factor (HIF) pathways [9]. Four major VEGF isoforms exist: 2 diffusive forms for intercellular signaling (VEGF-121 and VEGF-165) and 2 heparin binding heavier forms (VEGF-189 and VEGF-206) [10]. The cytokines promote secretion of matrix metalloproteinases that cut and activate [11] the VEGF-165 and possibly degrade the extracellular meshwork allowing heavier form to be released and then activated after a plasmin dependent cleavage.

The endothelial progenitor cells (EPCs) are attracted by the stromal cell derived factor 1-alpha (SDF1) that is known to be secreted by hypoxic or damaged retinal pigment epithelium (RPE) or retina [12, 13]. The only known receptor for SDF1 is CXCR4 that is expressed on the EPC and is responsible for their chemotaxis toward the damaged tissue. CXCR4 can also be expressed by some leukocytes that are involved in the membrane formation.

Guerin et al. [14] performed a detailed study on CNV of various etiologies, testing some of the most known hypothesis on this subject. There were some unavoidable biases in the study: for example, only advanced and partially fibrous membranes were collected, often unresponsive to the previous therapy. They suggest that RPE cells may play an important role in the development of CNV, the SDF1/CXCR4 axis is present in human, and there is a statistically significant association between detectable SDF1 and the neovascularization marker VEGFR-2.

Furthermore we performed an adjunctive statistical analysis on the dataset reported by Guerin: using Mann-Whitney test (in R environment [15]) we tested if immunohistochemical staining grading of the three main tissues (RPE, vascular network, and fibroblasts) for SDF1, CXCR4, and VEGFR-2 differs between inflammatory CNV and ARMD. In Table 1 the *P* values of the comparisons are reported. The study is underpowered for most of the comparisons but, interestingly, a low *P* value was found for the CXCR4 staining of the vascular meshwork of uveitis-related CNV versus ARMD-related CNV, suggesting that capillaries have a different role in the membrane development. Further studies on this distinctive aspect should be necessary.

CNV has also an extravascular component consisting in fibroblasts and leucocytes that express the CXCR4 themselves; furthermore RPE cells showed an increased production of tumor necrosis factor *α* (TNF*α*) and IL-1 [16] recruiting macrophages accounting for the inflammatory component of CNV, and also IL-2, IL-6, and IL-10 have been found, but their role is not clear yet [17].

Other mediators play a role in the membrane development: nitric oxide that induces the membrane formation, besides angiostatin, endostatin, CCR3, and the pigment epithelium derived growth factor (PEDF) contrasting the neovascularization. Focally the membrane can become fibrous and it is thought that the transforming growth factor *β* (TGF-*β*) is responsible for the process of recruiting choroidal fibroblasts, but on the other hand, at the same time, it induces the production of VEGF leading to the formation of new active foci [18].

## 4. Therapy and Clinical Studies

Understanding the uveitis as better as possible and identifying underlying infectious diseases are mandatory in order to keep the inflammation under control using the correct medical therapy. The use of steroids and immunosuppressors [19] has shown some utility in preventing and, sometimes, stopping the development of inflammatory CNV, but in the new millennium innovative therapies for ARMD came out and thus were tried on the inflammatory counterpart, leaving argon laser ablation, surgical membrane removal, and macular translocation a marginal role. But the uveitic subretinal membrane is less frequent than the wet ARMD, so researchers cannot freely design comparative studies.

In the literature most of clinical studies on inflammatory CNV therapy are case series with few underpowered retrospective studies often uncontrolled. Commonly patient selection was done in many different ways (naive/treated patients, active/quiescent uveitis, adult/pediatric, and different systemic therapy), making any attempt of rigorous meta-analysis impossible. We focused on the three main therapies available in the last decade: (i) photodynamic therapy (PDT), (ii) intravitreal bevacizumab (IVB), and (iii) intravitreal ranibizumab (IVR). We selected most important published articles in the last ten years with more than 2 subjects and, where possible, we extracted the data of patients. In Table 2 we report the name of the first author and the year of publication, the uveitis type included in the study, the median follow-up, where available or calculable, or the median follow-up time as provided in the paper. Moreover, we reported the number of subjects that after the treatment did not lose any line/letter on the total of patients, divided into three columns, one for each therapy, and the median numbers of injections performed or the mean numbers of injections if reported in the study. The articles are ordered by year of publication and then for first author name; at the end of the table we reported the sum and the percentages of success for each therapy in terms of visual acuity (VA) improvement and stabilization. We chose not to perform any statistical analysis on the data because such wide difference between the background studies could give highly biased results. Some well-known articles are not included because we could not extract the data about inflammatory patients only (as in Chang et al.) or because the dataset of patients resembled one of the other published articles by the same group of study.

The first articles report the case series on the PDT; overall success rate is quite high (78.4%) compared to previously reported significative vision loss in untreated patients (77% VA below 20/100) [51]. In most of these studies local or systemic steroid therapy was associated, and in two of them [25, 31] immunosuppressive drug was used in the majority of patients. Subsequently in the following years, the use of anti-VEGF therapy increased and IVB became available; 12 case series and 2 comparative retrospective studies about the IVB treatment in uveitis-related CNV are reported (Lott et al. [38] and Cornish et al. [45]). The first compares PDT to IVB in MC and the second IVB to IVR in PIC, but only in few cases. The work of Battaglia-Parodi did not show differences in overall success rate between the two therapies but showed a better visual recovery in patients treated with IVB. The final success rate for IVB seems to be around 87%. Finally in recent years IVR became more used, partly because of the concerns of the off-label use of IVB. We found the final success rate of the latter therapy to be around 90%, not very different by IVB treatment.

Although more than 30 articles were published about the argument, a decision about the treatment of inflammatory CNV cannot be assessed on evidence based medicine, as case series and uncontrolled studies are in the lower half of the scale of scientific evidences. Thus, well-designed randomized clinical trials should be necessary, but a correct comparison between the three main therapeutic strategies would need studies with a large number of people, which is not feasible for a rare complication of a rare disease such as posterior uveitis with strict inclusion and exclusion criteria.

A wise therapeutic approach we may suggest is the following:

- thorough control of the underlying inflammation using steroids, immunosuppressors, or specific treatment where appropriate;
- use of PDT for early extrafoveal lesions not causing a decrease in the VA, a less invasive procedure is always preferable in a uveitic eye in order to keep the possibility of flogosis reactivation low;
- use of IVR for foveal or juxtafoveal membranes or as second line therapy after PDT, the paper of Battaglia-Parodi demonstrated a higher VA for the IVB, but this drug is currently off-label for intravitreal use, and we could expect similar efficacy. Furthermore the literature showed that inflammatory CNV needs much less intravitreal injection than ARMD-related CNV to achieve the complete regression of the membrane.

Every year there is the announcement of new therapeutic approaches for wet ARMD, aflibercept, and stereotactic radiotherapy as examples, and the treatment of inflammatory CNV will benefit from these news although again it will be difficult to obtain a specific randomized controlled trial, so necessarily we will have to rely on indirect data.

## Figures and Tables

**Table 1:** *P* values of Mann-Whitney test performed on the dataset from Guerin et al. [14] comparing the staining grading for the three molecules studied (SDF1, CXCR4, and VEGFR-2) of the three structures of a CNV.

	SDF1			CXCR4			VEGFR-2		
	RPE	Vascularization	Fibroblasts	RPE	Vascularization	Fibroblasts	RPE	Vascularization	Fibroblasts
Inflammation versus ARMD	0.92	0.63	0.63	0.92	0.074	0.19	0.41	0.92	0.92

**Table 2:** Overview of the studies on the therapy of inflammatory CNV.

Study (year) [reference]	Uveitis type	FU	PDT	Bevacizumab (median numbers of injections)	Ranibizumab (median numbers of injections)
Saperstein et al. (2002) [20]	POHS	12	21/25		
Spaide et al. (2002) [21]	MC	10	7/7^§^		
Rogers et al. (2003) [22]	MISC	12	8/9^§^		
Wachtlin et al. (2003) [23]	MISC	22	17/19		
Nessi et al. (2004) [24]	TOXO	3	2/3^§^		
Leslie et al. (2005) [25]	MISC	11	6/6^§‡^		
Parodi et al. (2006) [26]	MC	12	6/7		
Coco et al. (2007) [27]	PIC	23	5/8^§^		
Gerth et al. (2006) [28]	MISC	23	7/14^§^		
Lim et al. (2006) [29]	MISC	12	3/5		
Mauget-Faÿsse (2006) [30]	TOXO	25	6/8		
Nowilaty and Bouhaimed (2006) [31]	VKH	19	4/6^§‡^		
Adán et al. (2007) [32]	MISC	7		8/9 (1)	
Chan et al. (2007) [33]	PIC	6		4/4 (3)	
Schadlu et al. (2008) [34]	POHS	6		26/28 (1.8*) most pts. had PDT	
Priyanka et al. (2009) [35]	MISC	15		4/6 (3)^§^	
Tran et al. (2008) [36]	MISC	6		10/10 (2.5)^§‡^	
Fine et al. (2009) [37]	MC	6		4/5 (1.5)	
Lott et al. (2009) [38]	MISC	7		15/21 (2)^§‡^	
Parodi et al. (2010) [39]	MC	12	9/13	12/14 (3.8*)	
Ehrlich et al. (2010) [40]	MISC	9	4/4^§^		
Kramer et al. (2010) [41]	MISC	12		10/10 (2)^§^	
Menezo et al. (2010) [42]	PIC	12			8/9 (1)^§^
Arevalo et al. (2011) [43]	MISC	12		21/23 (1)	
Carneiro et al. (2011) [44]	MISC	6			4/5 (3)
Cornish et al. (2011) [45]	PIC	12		5/6 (2)	2/3 (4)
Juliàn et al. (2011) [46]	MISC	15		12/15 (4.25*)^§‡^	
Rouvas et al. (2011) [47]	MISC	17			16/16 (2)
Troutbeck et al. (2012) [48]	MC	12			6/7 (3.4*)
Iannetti et al. (2013) [49]	MISC	19		7/8 (1)^§^	
Mansour et al. (2012) [50]	MISC	36		67/81 (3)	

Totals (median no of inj.) Percentual of success			105/134 78.4%	138/159 (2) 86.8%	36/40 (3) 90.0%

The first column shows the first author name, year of publication, and the reference in square brackets; the second column shows the type of uveitis studied (POHS: presumed ocular histoplasmosis, MC: multifocal choroiditis, MISC: miscellaneous, TOXO: toxoplasmosis, PIC: punctuate inner choroidopathy, and VKH: Vogt-Koyanagi-Harada disease); the third column shows the median follow-up calculated from dataset where not available; in the fourth, fifth, and sixth columns we reported the number of eyes whose VA stabilized or improved with the therapy over the number of eyes treated, respectively, for PDT, IVB, and IVR. Also we indicated the median numbers of injections needed or the mean number* if reported in the study. In the cells ^‡^indicates more than half patients had immunosuppressive treatment or ^§^for steroid therapy. The last row shows the number of cumulative successes in the eyes treated and the relative percentages. Further statistical analysis was impossible due to the extreme heterogeneity of the studies.

## References

[1] Baxter SL, Pistilli M, Pujari SS (2013). Risk of choroidal neovascularization among the uveitides. *American Journal of Ophthalmology*.

[2] Kuo IC, Cunningham ET (2000). Ocular neovascularization in patients with uveitis. *International Ophthalmology Clinics*.

[3] Moorthy RS, Chong LP, Smith RE, Rao NA (1993). Subretinal neovascular membranes in Vogt-Koyanagi-Harada syndrome. *American Journal of Ophthalmology*.

[4] Kotsolis AI, Killian FA, Ladas ID, Yannuzzi LA (2010). Fluorescein angiography and optical coherence tomography concordance for choroidal neovascularisation in multifocal choroidtis. *British Journal of Ophthalmology*.

[5] Sengupta N, Caballero S, Mames RN, Butler JM, Scott EW, Grant MB (2003). The role of adult bone marrow-derived stem cells in choroidal neovascularization. *Investigative Ophthalmology and Visual Science*.

[6] Sheridan CM, Rice D, Hiscott PS, Wong D, Kent DL (2006). The presence of AC133-positive cells suggests a possible role of endothelial progenitor cells in the formation of choroidal neovascularization. *Investigative Ophthalmology and Visual Science*.

[7] Gulati N, Forooghian F, Lieberman R, Jabs DA (2011). Vascular endothelial growth factor inhibition in uveitis: a systematic review. *British Journal of Ophthalmology*.

[8] Bhutto IA, McLeod DS, Hasegawa T (2006). Pigment Epithelium-Derived Factor (PEDF) and Vascular Endothelial Growth Factor (VEGF) in aged human choroid and eyes with age-related macular degeneration. *Experimental Eye Research*.

[9] Sheridan CM, Pate S, Hiscott P, Wong D, Pattwell DM, Kent D (2009). Expression of hypoxia-inducible factor-1*α* and -2*α* in human choroidal neovascular membranes. *Graefe’s Archive for Clinical and Experimental Ophthalmology*.

[10] Gitay-Goren H, Soker S, Vlodavsky I, Neufeld G (1992). The binding of vascular endothelial growth factor to its receptors is dependent on cell surface-associated heparin-like molecules. *Journal of Biological Chemistry*.

[11] Lee S, Jilan SM, Nikolova GV, Carpizo D, Luisa Iruela-Arispe M (2005). Processing of VEGF-A by matrix metalloproteinases regulates bioavailability and vascular patterning in tumors. *Journal of Cell Biology*.

[12] Bhutto IA, McLeod DS, Merges C, Hasegawa T, Lutty GA (2006). Localisation of SDF-1 and its receptor CXCR4 in retina and choroid of aged human eyes and in eyes with age related macular degeneration. *British Journal of Ophthalmology*.

[13] Butler JM, Guthrie SM, Koc M (2005). SDF-1 is both necessary and sufficient to promote proliferative retinopathy. *Journal of Clinical Investigation*.

[14] Guerin E, Sheridan C, Assheton D (2008). SDF1-alpha is associated with VEGFR-2 in human choroidal neovascularisation. *Microvascular Research*.

[15] R Core Team R: a language and environment for statistical computing. http://www.R-project.org/.

[16] Crane IJ, Wallace CA, McKillop-Smith S, Forrester JV (2000). CXCR4 receptor expression on human retinal pigment epithelial cells from the blood-retina barrier leads to chemokine secretion and migration in response to stromal cell-derived factor 1*α*. *Journal of Immunology*.

[17] Izumi-Nagai K, Nagai N, Ozawa Y (2007). Interleukin-6 receptor-mediated activation of signal transducer and activator of transcription-3 (STAT3) promotes choroidal neovascularization. *American Journal of Pathology*.

[18] Bian Z-M, Elner SG, Elner VM (2007). Regulation of VEGF mRNA expression and protein secretion by TGF-*β*2 in human retinal pigment epithelial cells. *Experimental Eye Research*.

[19] Dees C, Arnold JJ, Forrester JV, Dick AD (1998). Immunosuppressive treatment of choroidal neovascularization associated with endogenous posterior uveitis. *Archives of Ophthalmology*.

[20] Saperstein DA, Rosenfeld PJ, Bressler NM (2002). Photodynamic therapy of subfoveal choroidal neovascularization with verteporfin in the ocular histoplasmosis syndrome: one-year results of an uncontrolled, prospective case series. *Ophthalmology*.

[21] Spaide RF, Freund KB, Slakter J, Sorenson J, Yannuzzi LA, Fisher Y (2002). Treatment of subfoveal choroidal neovascularization associated with multifocal choroiditis and panuveitis with photodynamic therapy. *Retina*.

[22] Rogers AH, Duker JS, Nichols N, Baker BJ (2003). Photodynamic therapy of idiopathic and inflammatory choroidal neovascularization in young adults. *Ophthalmology*.

[23] Wachtlin J, Heimann H, Behme T, Foerster MH (2003). Long-term results after photodynamic therapy with verteporfin for choroidal neovascularizations secondary to inflammatory chorioretinal diseases. *Graefe’s Archive for Clinical and Experimental Ophthalmology*.

[24] Nessi F, Guex-Crosier Y, Ambresin A, Zografos L (2004). Photodynamic therapy with verteporfin for subfoveal choroidal neovascularization secondary to toxoplasmic chorioretinal scar. *Klinische Monatsblatter fur Augenheilkunde*.

[25] Leslie T, Lois N, Christopoulou D, Olson JA, Forrester JV (2005). Photodynamic therapy for inflammatory choroidal neovascularisation unresponsive to immunosuppression. *British Journal of Ophthalmology*.

[26] Parodi MB, Iacono P, Spasse S, Ravalico G (2006). Photodynamic therapy for juxtafoveal choroidal neovascularization associated with multifocal choroiditis. *American Journal of Ophthalmology*.

[27] Coco RM, De Souza CF, Sanabria MR (2007). Photodynamic therapy for subfoveal and juxtafoveal choroidal neovascularization associated with punctate inner choroidopathy. *Ocular Immunology and Inflammation*.

[28] Gerth C, Spital G, Lommatzsch A, Heiligenhaus A, Pauleikhoff D (2006). Photodynamic therapy for choroidal neovascularization in patients with multifocal choroiditis and panuveitis. *European Journal of Ophthalmology*.

[29] Lim JI, Flaxel CJ, LaBree L (2006). Photodynamic therapy for choroidal neovascularisation secondary to inflammatory chorioretinal disease. *Annals of the Academy of Medicine Singapore*.

[30] Mauget-Faÿsse M, Mimoun G, Ruiz-Moreno JM (2006). Verteporfin photodynamic therapy for choroidal neovascularization associated with toxoplasmic retinochoroiditis. *Retina*.

[31] Nowilaty SR, Bouhaimed M (2006). Photodynamic therapy for subfoveal choroidal neovascularisation in Vogt-Koyanagi-Harada disease. *British Journal of Ophthalmology*.

[32] Adán A, Mateo C, Navarro R, Bitrian E, Casaroli-Marano RP (2007). Intravitreal bevacizumab (Avastin) injection as primary treatment of inflammatory choroidal neovascularization. *Retina*.

[33] Chan W-M, Lai TYY, Liu DTL, Lam DSC (2007). Intravitreal bevacizumab (Avastin) for choroidal neovascularization secondary to central serous chorioretinopathy, secondary to punctate inner choroidopathy, or of idiopathic origin. *American Journal of Ophthalmology*.

[34] Schadlu R, Blinder KJ, Shah GK (2008). Intravitreal bevacizumab for choroidal neovascularization in ocular histoplasmosis. *American Journal of Ophthalmology*.

[35] Priyanka P, Bhat P, Sayed R, Foster CS (2009). Intravitreal bevacizumab for uveitic choroidal neovascularization. *Ocular Immunology and Inflammation*.

[36] Tran THC, Fardeau C, Terrada C, Ducos De Lahitte G, Bodaghi B, Lehoang P (2008). Intravitreal bevacizumab for refractory choroidal neovascularization (CNV) secondary to uveitis. *Graefe’s Archive for Clinical and Experimental Ophthalmology*.

[37] Fine HF, Zhitomirsky I, Freund KB (2009). Bevacizumab (avastin) and ranibizumab (lucentis) for choroidal neovascularization in multifocal choroiditis. *Retina*.

[38] Lott MN, Schiffman JC, Davis JL (2009). Bevacizumab in inflammatory eye disease. *American Journal of Ophthalmology*.

[39] Parodi MB, Iacono P, Kontadakis DS, Zucchiatti I, Cascavilla ML, Bandello F (2010). Bevacizumab vs photodynamic therapy for choroidal neovascularization in multifocal choroiditis. *Archives of Ophthalmology*.

[40] Ehrlich R, Kramer M, Rosenblatt I (2010). Photodynamic therapy for choroidal neovascularization in young adult patients. *International Ophthalmology*.

[41] Kramer M, Axer-Siegel R, Jaouni T (2010). Bevacizumab for choroidal neovascularization related to inflammatory diseases. *Retina*.

[42] Menezo V, Cuthbertson F, Downes SM (2010). Positive response to intravitreal ranibizumab in the treatment of choroidal neovascularization secondary to punctate inner choroidopathy. *Retina*.

[43] Arevalo JF, Adan A, Berrocal MH (2011). Intravitreal bevacizumab for inflammatory choroidal neovascularization: results from the Pan-American collaborative retina study group at 24 months. *Retina*.

[44] Carneiro AM, Silva RM, Veludo MJ (2011). Ranibizumab treatment for choroidal neovascularization from causes other than age-related macular degeneration and pathological myopia. *Ophthalmologica*.

[45] Cornish KS, Williams GJ, Gavin MP, Imrie FR (2011). Visual and optical coherence tomography outcomes of intravitreal bevacizumab and ranibizumab in inflammatory choroidal neovascularization secondary to punctate inner choroidopathy. *European Journal of Ophthalmology*.

[46] Julián K, Terrada C, Fardeau C (2011). Intravitreal bevacizumab as first local treatment for uveitis-related choroidal neovascularization: long-term results. *Acta Ophthalmologica*.

[47] Rouvas A, Petrou P, Douvali M (2011). Intravitreal ranibizumab for the treatment of inflammatory choroidal neovascularization. *Retina*.

[48] Troutbeck R, Bunting R, van Heerdon A, Cain M, Guymer R (2012). Ranibizumab therapy for choroidal neovascularization secondary to non-age-related macular degeneration causes. *Clinical and Experimental Ophthalmology*.

[49] Iannetti L, Paroli MP, Fabiani C (2013). Effetcts of intravitreal Bevacizumab on inflammatory choroidal neovascular membrane. *European Journal of Ophthalmology*.

[50] Mansour AM, Arevalo JF, Fardeau C (2012). Three-year visual and anatomic results of administrating intravitreal bevacizumab in inflammatory ocular neovascularization. *Canadian Journal of Ophthalmology*.

[51] Brown J, Folk JC, Reddy CV, Kimura AE (1996). Visual prognosis of multifocal choroiditis, punctate inner choroidopathy, and the diffuse subretinal fibrosis syndrome. *Ophthalmology*.

